# Phytosterols and Benign Prostatic Hyperplasia Management: Knowledge and Perceptions Among Future Healthcare Professionals

**DOI:** 10.7759/cureus.72083

**Published:** 2024-10-21

**Authors:** Mădălina-Georgiana Buț, Amalia Puscas, George Jitca, Tudor-Ionuț Istrate, Amelia Tero-Vescan

**Affiliations:** 1 Biochemistry, George Emil Palade University of Medicine, Pharmacy, Science, and Technology of Târgu Mureș, Târgu Mureș, ROU; 2 Pharmacology and Clinical Pharmacy, George Emil Palade University of Medicine, Pharmacy, Science, and Technology of Târgu Mureș, Târgu Mureș, ROU

**Keywords:** benign prostatic hyperplasia, complementary therapies, dietary supplements, future healthcare professionals, phytosterols

## Abstract

Background

Benign prostatic hyperplasia (BPH) is a common condition in elderly men, with a growing global incidence. Dietary supplements containing phytosterols (DS-PS) are increasingly used as complementary treatments for BPH. However, there is limited understanding of the knowledge and perceptions future healthcare professionals have regarding these supplements, which may affect the quality of medical counseling and patient care.

Aim

The present study aims to obtain a clear and detailed understanding of the level of knowledge and existing perceptions among medical students regarding DS-PS used in BPH.

Materials and methods

A structured questionnaire based on 18 questions was distributed to students from the medicine and pharmacy programs of the George Emil Palade University of Medicine, Pharmacy, Science, and Technology of Târgu Mureș, Romania. Descriptive statistics was applied to all the questions, and potential associations were evaluated by the chi-square test.

Results

The study included 361 participants, predominantly female (54.61%) and aged between 18 and 25 years (82.06%). Although 80.43% of respondents were familiar with the concept of BPH, only 57.99% knew that phytosterols (PS) are used to improve the symptoms associated with BPH. A significant finding is that pharmacy students had a higher level of knowledge about DS-PS (79.77%) compared to medical students (54.49%). However, both groups show similar levels of general knowledge about BPH. Additionally, there was a significant association between the awareness of DS-PS and the perception of their safety, as well as between the level of knowledge about BPH and the understanding of the benefits offered by PS in the treatment of BPH (p<0.05).

Conclusion

This study shows a positive perception of PS among respondents, even in the absence of detailed knowledge, highlighting the need for integrative curricula for medical students and advanced training for healthcare professionals focused on the use of complementary phytotherapeutic approaches in chronic conditions. Integrating this information into university curricula and promoting evidence-based information could enhance clinical practice and patient counseling.

## Introduction

Benign prostatic hyperplasia (BPH), also known as prostate gland enlargement, is one of the most common conditions affecting men as they age. According to autopsy studies, the prevalence of this condition varies with age, being estimated at 8% around the age of 40, 50% around the age of 60, and 80% around the age of 80. Moreover, the incidence of BPH is rising significantly worldwide, particularly in low- and middle-income countries undergoing significant demographic and epidemiological changes [1,2]. Recent studies confirm previous findings. A 2024 study by Ye et al. reports a significant global increase in BPH cases over the past three decades, with prevalent and incident cases rising by 118.78% and 121.22%, respectively. By 2019, 79 million people aged 60 and older were affected. The highest burden was observed in China, India, and the U.S., with the highest prevalence in Eastern Europe, Central, and Andean Latin America. The increase is primarily attributed to population growth (94.93%), epidemiological changes (3.45%), and aging (1.62%) [3]. This trend underscores the need for greater attention and a well-thought-out strategy to prevent overburdening healthcare systems and to successfully manage the symptoms associated with BPH.

In pharmaceutical practice, the conventional therapy for BPH includes 5-alpha reductase inhibitors, which block the conversion of testosterone into dihydrotestosterone, the main factor involved in prostate growth, and selective alpha-receptor blockers, which relax the smooth muscles of the prostate and bladder, facilitating urinary flow and alleviating symptoms. These prescription-only drugs, recommended in advanced stages of BPH often cause sexual dysfunctions, orthostatic hypotension, dizziness, headaches, and fatigue [4-6]. Consequently, many patients initially turn to dietary supplements (DS), particularly plant-based ones. DS-containing phytosterols (DS-PS) are the most commonly used as a symptomatic treatment of BPH. Phytosterols (PS) show promising clinical applicability as an adjuvant therapy in BPH due to their beneficial effects on lower urinary tract symptoms and the proliferation of prostatic cells. The primary mechanism involved is the inhibition of the 5-alpha-reductase, the enzyme responsible for dihydrotestosterone production. Additionally, the positive effects of PS on BPH have been demonstrated in both preclinical and clinical studies, which highlight their pro-apoptotic, anti-inflammatory, and antioxidant properties [7-9].

Although evidence exists supporting their potential beneficial effects [7-9], the European Association of Urology International (EUA) and American Urological Association (AUA) guidelines do not support the use of DS due to inconsistencies and lack of uniformity in the available studies [4,5]. Their application in clinical practice remains limited, with such supplements often being taken at the patient’s initiative without the recommendation or guidance of a physician or pharmacist. Consequently, based on our collective professional experience in clinical practice, practitioners tend to focus more on conventional therapies rather than on the use of phytotherapeutic compounds. The lack of trust is mainly caused by the discrepancy between the quality standards applied to prescription drugs compared to DS [10]. Additionally, potential interactions with other medications can occur due to gaps in healthcare professionals' education or limited research funding allocated to this field. Numerous studies in the literature emphasize that academic training is directly correlated with clinical performance, highlighting the importance of solid curricula in preparing practitioners to apply theoretical knowledge in medical practice [11-13].

Currently, there are no studies in the literature that directly evaluate the knowledge level and perceptions of health science students regarding the use of DS-PS in BPH. Therefore, this study aims to raise awareness of the importance of complementary and alternative treatments in managing BPH symptoms. DS-PS represents a relatively new and less studied, but potentially effective, option that can support standard drug therapies.

This research focuses on several specific objectives such as assessing knowledge regarding BPH, analyzing the attitude of the studied population toward the use of DS-PS in BPH and the benefits they provide, evaluating awareness related to the safety of these supplements, as well as the circumstances under which respondents became familiar with DS-PS.

Adequate education of current and future healthcare professionals about these topics can encourage the transfer of knowledge to the patients, thereby improving the quality of the medical act. The results of the questionnaire may highlight potential gaps in medical education programs and provide valuable insights for updating and improving the curriculum, ensuring that students are well-informed about complementary treatment options for BPH.

## Materials and methods

Study design

This descriptive cross-sectional study aimed to evaluate the knowledge and perceptions of future healthcare professionals regarding the use of DS-PS in alleviating symptoms associated with BPH. The study was conducted through a structured questionnaire distributed to students from the pharmacy and medicine programs of the George Emil Palade University of Medicine, Pharmacy, Science, and Technology of Târgu Mureș, Târgu Mureș, Romania.

Participants

A total of 361 valid responses were obtained between June and July 2024. According to sample size calculations, a minimum sample of 345 students was required to ensure a statistical power of 95% and a two-sided significance level of 5% (i.e., a two-sided p-value of less than 0.05). The sample size was determined using the Raosoft online sample size calculator (Raosoft Inc., Seattle, USA) [14]. The study included only students enrolled in the medicine and pharmacy programs of the George Emil Palade University of Medicine, Pharmacy, Science, and Technology of Târgu Mureș. Exclusion criteria included individuals who are not currently enrolled in the medicine or pharmacy programs at the university, participants who did not complete the entire questionnaire or provided inconsistent answers, and students from other academic programs or faculties who were excluded because they did not fit the study's target population.

Questionnaire

A self-structured questionnaire, consisting of 18 questions, was prepared and used to collect the data (Table 1). The first section gathers demographic information to categorize respondents by age, gender, and education (Q1-4). The second section (Q5-8) evaluates the participants' knowledge of BPH and their understanding of the use of PS-DS in the context of BPH. This is followed by questions (Q9-11) that assess specific knowledge about PS (category, source, mechanism) and their implications in BPH, aiming to evaluate the students' foundational understanding of the condition and related treatments. The subsequent section (Q12-15) examines respondents' views on the effectiveness, safety, and dispensing practices of these supplements in pharmacies. Finally, the questionnaire assesses how important respondents consider PS in their field of study (Q16) and whether they have encountered related scientific discussions (Q17-18).

**Table 1:** Assessment of knowledge and attitudes regarding DS-PS in BPH. This table constitutes the full questionnaire DS-PS: dietary supplements based on phytosterols; BPH: benign prostatic hyperplasia

No.	Question	Response options
1	To which age group do you belong?	□ 18-25 □ 25-34 □ 35-44 □ 45-59 □ Over 59
2	Please specify your gender:	□ Female □ Male
3	Which higher education programs have you completed since finishing high school?	□ Bachelor’s degree program (please specify faculty/specialization) □ Master’s degree program (please specify faculty/specialization) □ Doctoral degree program (please specify field) □ Other educational programs (please specify) □ Not applicable
4	What faculty and specialization are you currently studying?	□ Pharmacy (please specify specialization and year) □ Medicine (please specify specialization and year) □ Other (please specify) □ Not applicable
5	Are you familiar with the term prostate adenoma, also called benign prostatic hyperplasia?	□ Yes □ No
6	How would you assess your knowledge of benign prostatic hyperplasia on a scale of 1 to 5?	□ 1 □ 2 □ 3 □ 4 □ 5
7	Had you heard of phytosterol-based dietary supplements prior to this questionnaire?	□ Yes □ No
8	Did you know that phytosterols can be used as a dietary supplement for benign prostatic hyperplasia?	□ Yes □ No
9	What are phytosterols? (Select all that apply)	□ Plant-derived compounds □ Synthetic compounds □ Used in dietary supplements □ Used in pharmaceutical medications □ I don't know
10	Indicate the context in which you learned about the use of phytosterol-based dietary supplements in treating benign prostatic hyperplasia:	□ University courses □ Specialty books □ Internship in hospital/pharmacy/medical office □ Advertisements on the internet or TV/mass media □ Pharmacies/supermarkets/health food stores □ Other sources □ Not applicable
11	If you learned about phytosterol-based dietary supplements at university, please specify the year of study in which you learned about them:	□ Year 1 □ Year 2 □ Year 3 □ Year 4 □ Year 5 □ Year 6
12	How do you believe phytosterols contribute to managing benign prostatic hyperplasia? (Select all applicable options)	□ Reduce inflammation □ Lower cholesterol levels □ Inhibit growth of prostate cells □ Increase urinary flow □ I don’t know
13	Do you think phytosterol-based dietary supplements provide benefits for patients with benign prostatic hyperplasia?	□ Yes, as an adjuvant to medical treatment □ Yes, they may effectively replace medical treatment □ No, I don't believe they bring real benefits □ I have no knowledge on this subject
14	Do you consider phytosterol-based dietary supplements to be safe for consumers?	□ They are not safe □ Yes, they are safe □ They are safe only if the recommended daily dose is not exceeded □ I have no knowledge on this subject
15	Do you think that phytosterol-based dietary supplements should be dispensed from pharmacies:	□ Upon doctor's recommendation □ Upon pharmacist's recommendation □ Upon patient's request □ I'm not sure □ I don't know
16	How relevant do you consider understanding the effects of phytosterol-based dietary supplements in your field of study or professional activity?	□ Not relevant □ Slightly relevant □ Moderately relevant □ Highly relevant
17	Have you attended any scientific discussions or presentations regarding phytosterol-based dietary supplements?	□ Yes □ No
18	If yes, in what context?	□ University courses □ Specialty books □ Practical training □ Advertisements on the internet or TV/mass media/radio □ Other sources

The content validity was assessed by consulting experts in pharmacology and urology to ensure that the questions were both relevant and appropriate. The questionnaire was initially distributed to professors from the community medicine and pharmacy programs, as well as to postgraduate students, to verify whether the questions were clear, direct, and easy to understand. Based on the feedback received, minor revisions were made to improve the precision of the questions and eliminate any ambiguities. A pilot test was then conducted with a small sample of students to further validate the questionnaire, ensuring that all items were well understood and aligned with the study's objectives. The feedback obtained from the pilot test was used to make final adjustments before distributing the questionnaire to the entire study population.

Data collection

The questionnaire was distributed to the target population through online platforms as well as physical surveys over a period of two months (June-July 2024). Participation was anonymous, and all participants provided informed consent prior to completing the questionnaire.

Ethical considerations

This study was approved by the Scientific Research Ethics Committee of George Emil Palade University of Medicine, Pharmacy, Science, and Technology of Târgu Mureș (approval number: 3254/17.06.2024). All procedures adhered to the ethical standards of the institution and followed the Declaration of Helsinki guidelines. Participation was voluntary, and anonymity was maintained throughout the study.

Statistical analysis

Descriptive parameters were calculated for all the survey questions to characterize the responses. The chi-square test was used to evaluate potential associations between variables, such as the study program and knowledge of BPH, the relationship between PS and BPH, gender and knowledge of BPH and PS-DS, as well as the use of PS in BPH and awareness of PS-DS and perceptions of their safety. A p-value of less than 0.05 was considered statistically significant. All statistical analyses were conducted using GraphPad Prism (version 9, GraphPad Software, San Diego, USA).

## Results

Sociodemographic characteristics

This study included 361 participants who completed the questionnaire, all of whom were students in the medicine and pharmacy programs at the George Emil Palade University of Medicine, Pharmacy, Science, and Technology of Târgu Mureș. The majority of respondents were female (54.61%), with the remaining 45.38% being male. The largest proportion of participants fell within the 18-25 age group (82.06%). Of all the respondents, the majority are studying medicine (75.81%), while the rest are enrolled in the pharmacy program (24.18%) (Table 2).

**Table 2:** Sociodemographic characteristics of the study participants N: number of participants in each category

Sociodemographic characteristics of the study participants (n=361)			
Variables		N	Percentage (%)
Gender	Female	201	54.61
	Male	167	45.38
Age	18-25	302	82.06
	25-34	55	14.94
	35-44	7	1.9
	45-59	4	1.09
Ongoing studies	Medicine	279	75.81
	Pharmacy	89	24.18

General knowledge of BPH

**Figure 1:**
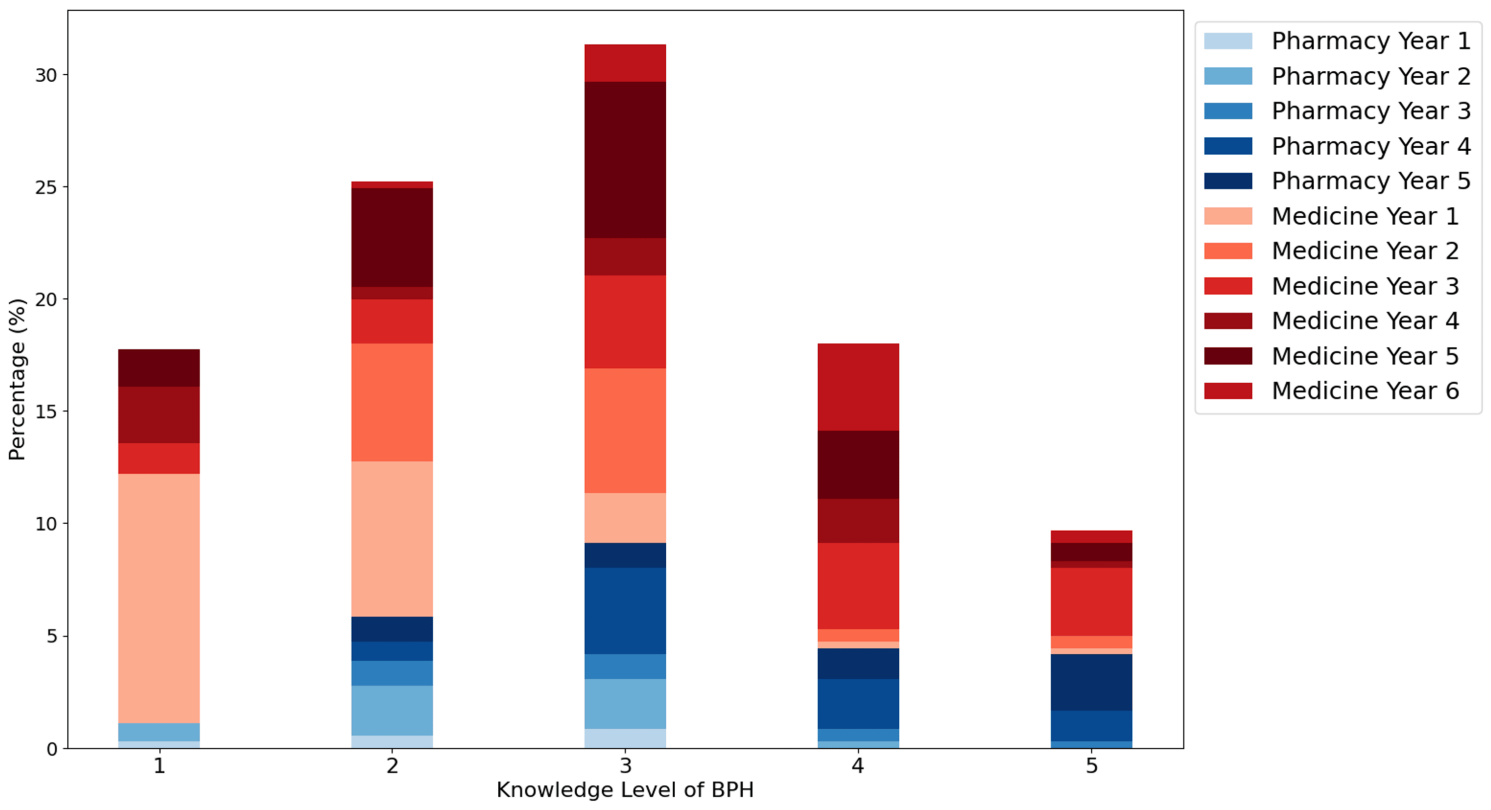
Distribution of responses based on BPH knowledge level, specialization, and year of study The histogram shows the distribution of BPH knowledge on a scale from 1 to 5 (with 1 being the lowest level of knowledge and 5 the highest); the color of the bars indicates different specializations and years of study: pharmacy (years 1-5) and medicine (years 1-6). BPH: benign prostatic hyperplasia

The assessment of respondents' level of awareness regarding general concepts of BPH shows that the majority rated their knowledge as moderate at levels 2 and 3, with only a small percentage of 9.51% rating their knowledge as very high (5) (Figure 1). Senior years in the medicine program (years 5 and 6) and in the pharmacy program (year 5) are predominantly represented at higher knowledge levels (4 and 5), suggesting that senior students tend to have a deeper understanding of BPH. These observations indicate that, in general, the level of knowledge about BPH increases as students progress through their years of study, particularly in the medical program.

Knowledge of DS-PS and their connection with BPH

Regarding respondents' knowledge about the connection between BPH and DS-PS in BPH, women were found to be more familiar with these concepts. The percentages of women who reported having knowledge about BPH and DS-PS are 81.59%, 65.67%, and 38.30%, compared to men, where the respective percentages are 76.04%, 62.87%, and 32.92% (Table 3). However, despite the percentage difference, statistical tests indicate that there is no significant difference between the number of women and men who are well aware of BPH and DS-PS used in BPH (p>0.05).

**Table 3:** Distribution of positive responses to the questions *BPH: awareness of the concept of BPH; **DS-PS: knowledge regarding DS-PS; ***DS-PS-BPH: awareness of the relationship between DS-PS and BPH BPH: benign prostatic hyperplasia; DS: dietary supplements; PS: phytosterols; DS-PS: dietary supplements based on phytosterols; N: number of participants in each category

Associations between demographic characteristics and knowledge of BPH and DS-PS used in BPH							
		*BPH		**DS-PS		***DS-PS-BPH	
		N	%	N	%	N	%
Gender	Female	164	81.59	132	65.67	77	38.30
	Male	127	76.04	105	62.87	60	35.92
Ongoing studies	Medicine	220	78.85	166	54.49	86	30.82
	Pharmacy	71	79.77	71	79.77	47	52.80

According to the present study, 78.85% of medical students are familiar with the concept of BPH, 54.49% are aware of DS-PS, and only 30.82% know that DS-PS is used in BPH. In comparison, pharmacy students show the following percentages: 79.77% for the BPH concept, 79.77% regarding DS-PS, and 52.80% for the use of DS-PS in BPH, respectively. Statistically, the results indicate a significant difference between medical and pharmacy students regarding their knowledge of DS-PS and their use in BPH, with pharmacy students demonstrating a higher level of knowledge (p<0.05). The data presented suggest that pharmacy students tend to have a higher level of knowledge about DS-PS and their connection to BPH compared to medical students. As for general knowledge about BPH, medical students have relatively similar percentages to those of pharmacy students.

Specific knowledge about DS-PS

**Figure 2:**
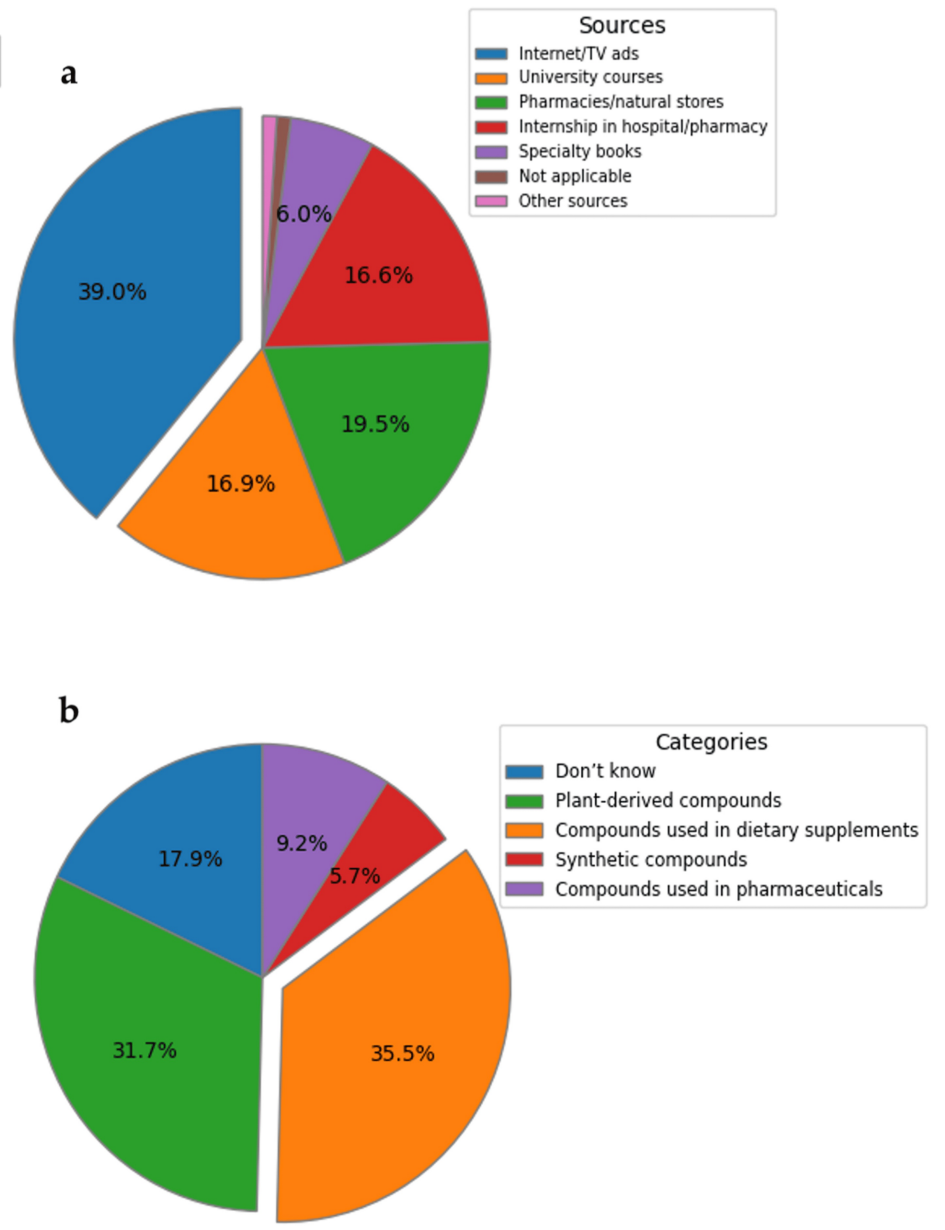
Distribution of study participants' responses regarding their perceptions of the source and category of PS Distribution of responses regarding (a) the sources of information about PS (question 10) and (b) the category to which PS belongs (question 9); all the questions can be viewed in Table 1. PS: phytosterols

The analysis of responses regarding specific knowledge about DS-PS (Figure 2) reveals that the majority of respondents are aware that PS are plant-derived compounds (35.5%) used in the formulation of DS (31.7%), while a significant segment is unsure about these categories (17.9%) (Figure 2b). The primary sources of information are the Internet and TV/media advertisements (39.0%), indicating a major impact of mass media (Figure 2a).

**Figure 3:**
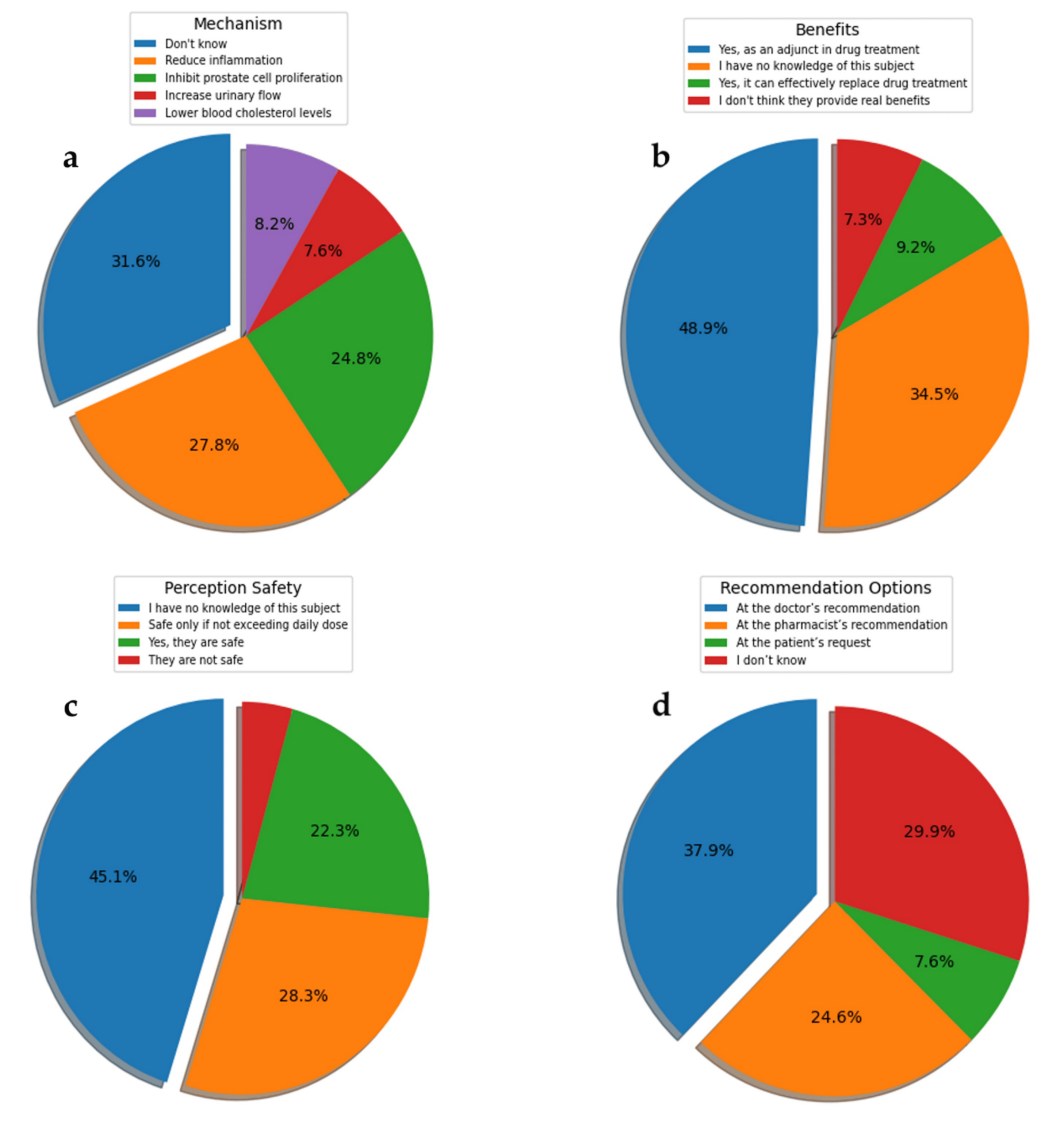
Distribution of study participants' responses regarding specific knowledge about DS-PS Distribution of responses regarding (a) the mechanism of action, (b) the benefits, (c) perception of safety, and (d) recommendation of PS (questions 12, 13, 14, and 15); all the questions can be viewed in Table 1. DS-PS: dietary supplements based on phytosterols

Regarding the mechanisms of action of PS, knowledge of their anti-inflammatory effects (27.8%) and inhibition of prostate cell proliferation (24.8%) is predominant, although a significant percentage of respondents are unaware of the mechanism of action (31.6%) (Figure 3a). The analysis of Figure 3b shows that 48.9% of respondents believe that PS is beneficial as an adjuvant in drug treatments, while 34.5% do not have sufficient knowledge on this topic. Regarding safety (Figure 3c), 45.1% of respondents are unaware of this aspect, 28.3% believe they are safe only if the patients use the recommended dose, 22.3% believe they are safe in any situation, and only a small percentage (4.3%) consider that PS is not safe for administration. The chi-square test shows a significant association between awareness of DS-PS and the perception of their safety (p<0.05). Most respondents believe that PS should be recommended by a physician (37.9%), or a pharmacist (24.6%), while some have no clear opinion (29.9%), or believe they should be dispensed at the patient's request (7.6%) (Figure 3d).

Respondents were asked about the relevance of knowing the effects of DS-PS in their field of study or work. The distribution of responses showed that 28.53% consider this knowledge moderately relevant, while 26.09% view it as irrelevant. When asked about their participation in discussions or scientific presentations on PS, 82.07% responded negatively. Among those who participated, the majority (80.30%) gained their information through university courses or specialty books.

**Figure 4:**
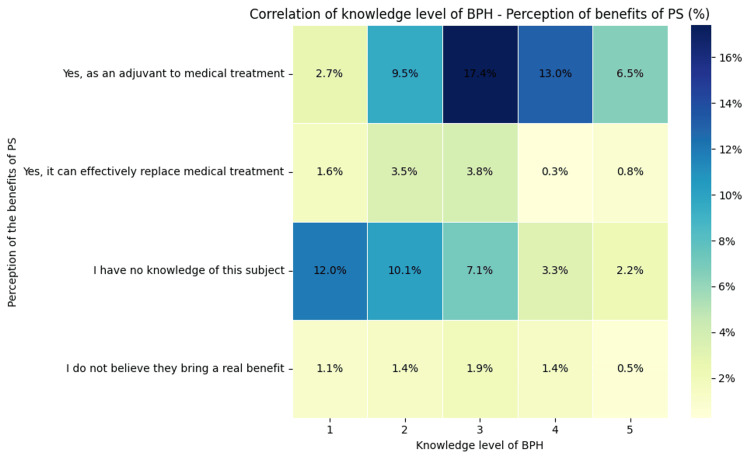
Correlation between the level of knowledge about BPH and the perception of the benefits of PS in BPH BPH: benign prostatic hyperplasia; PS: phytosterols

On the other hand, a possible correlation between the level of knowledge about BPH and the perception of the benefits provided by PS was tested. Figure 4 below shows that the response "PS is beneficial as adjuvant to medical treatment" increases with the level of BPH knowledge. For example, at a BPH knowledge level of "3," 17.4% of respondents consider this, compared to only 2.7% at level "1." A significant proportion of respondents (e.g., 12.0% at level "1") report that they have no knowledge of DS-PS. This proportion decreases as the level of BPH knowledge increases (reaching 2.2% at level "5"). The perception that PS does not offer real benefits is relatively low across all categories, ranging from 1.1% at level "1" to 0.5% at level "5."

## Discussion

The results of our study indicate that the level of knowledge among health science students regarding BPH and DS-PS varies significantly, with notable differences between medical and pharmacy students. This variability can be partially explained by the different curricula, which focus more on bioactive substances and their use in treatments for pharmacy students, while medical students seem to have a deeper understanding of the clinical aspects of BPH.

This finding is consistent with other studies in the literature, which emphasize that future healthcare professionals tend to have a stronger knowledge base in conventional therapies compared to DS and phytotherapy. For example, a study conducted by Ventola highlighted that most medical students have a limited level of knowledge about DS and generally perceive herbal supplements as less effective and less safe compared to conventional medications. Additionally, Ventola's study emphasizes that pharmacists are in an ideal position to provide counseling and monitor patients' use of these therapies. However, they note that this lack of engagement from pharmacists and other healthcare professionals is partly due to a lack of the knowledge, confidence, and training necessary to provide proper guidance regarding complementary therapies [15].

Out of a total of 361 respondents, a significant 80.43% are familiar with the concept of BPH. This suggests that the majority of respondents have a basic level of knowledge about BPH, which is a positive indicator within a university audience. However, among those familiar with the concept of BPH, only 73.98% have heard of DS-PS, raising questions about the level of awareness and understanding of the role these supplements play in BPH treatment. Furthermore, only 57.99% of those aware of PS knew they were used in the treatment of BPH. Although most respondents are familiar with the concept of BPH, there is a significant gap in specific knowledge about DS-PS-BPH.

Furthermore, another important aspect is related to the source of information, as a significant percentage (39.0%) reported "internet/TV or mass media advertisements" as their primary source of information, indicating that the selected sample tends to obtain their information about DS more from commercial sources rather than academic or medical ones. Other studies in the literature highlight that advertisements and marketing are among the main sources of information for consumers, underscoring a deficit in evidence-based information [16-18]. This raises concerns about the accuracy and quality of the information received, as advertisements are often sales-oriented and less focused on proper education. This may indicate a generally positive perception or acceptance of PS among respondents, even in the absence of advanced medical knowledge.

The previously obtained results are confirmed by responses to the question regarding participation in discussions or scientific presentations about DS-PS, whether in academic research or other scientific contexts, where only 17.93% of participants responded affirmatively. Additionally, a significant percentage (26.9%) consider this subject irrelevant to their field of work. These results can also be explained by the lack of investment in DS research, with funding often being limited and most studies conducted by PhD students or researchers in the early stages of their careers [19,20].

The graph in Figure 3, illustrating the correlation between the level of knowledge about BPH and the perception of the benefits of PS, suggests that as knowledge about BPH increases, respondents become more confident in the benefits of PS as adjuvants in drug treatments and are less likely to report having no knowledge on the subject. Additionally, those with more in-depth knowledge are more cautious about considering PS as a complete replacement for drug treatments. Furthermore, there is a statistically significant association between awareness of the use of PS in BPH and opinions about their safety. In other words, respondents' views on the safety of PS are linked to whether they know that PS is used in BPH. These trends highlight the importance of education and information in shaping perceptions about the use of PS [16-18].

As a result, it can be said that students' level of knowledge directly influences their ability to provide quality care, make informed clinical decisions, and contribute to the improvement of clinical practice through continuous education and research. These findings are supported by the study conducted by Posadzki et al., which shows that adequate education and training in phytotherapy can improve the perception and confidence of future professionals in the use of complementary and alternative treatments. This suggests that better integration of phytotherapy and evidence-based information about DS into university curricula could enhance the understanding and application of these therapies in clinical practice [21].

This study presents several limitations that need to be taken into account when interpreting the results. First, the use of a convenience sampling method may limit the generalizability of the findings, as the sample was drawn only from students at a single university, which may not fully represent all healthcare students, especially considering that curricula can vary slightly from one university to another. Furthermore, the cross-sectional design captures knowledge and perceptions at a single moment in time, limiting the ability to evaluate how these views may evolve with further education or professional experience. It would have also been beneficial to compare the knowledge and perceptions of students, who only have theoretical knowledge, with those of graduates who also have practical experience. Future research should aim to address these limitations by incorporating a larger and more diverse sample, a longitudinal design, and alternative methods of data collection.

## Conclusions

The study's data may indicate a generally positive perception or acceptance of PS among respondents, even in the absence of advanced medical knowledge. The results of this study highlight the need for additional educational efforts to improve the knowledge level of future healthcare professionals regarding DS-PS and their use in the treatment of BPH. Furthermore, it is imperative to conduct more robust and standardized studies, similar to those performed for current pharmaceuticals, to accurately evaluate the efficacy of these phytotherapeutic strategies. These studies should be based on rigorous methodologies and conducted through long-term multicenter trials using an appropriate placebo. It is also essential to maintain consistent dosing and apply validated techniques for the extraction of biologically active compounds. Such an approach would yield more reliable and comparable data, enabling a clearer understanding of the potential therapeutic benefits of these DS, particularly in clinical settings.

A deeper integration of this information into university curricula, combined with more effective promotion of evidence-based information, could contribute to improving clinical practice and providing more informed advice to patients.
